# Advanced 3D Insights Into the Marginal and Internal Fit of Ceramic‐Filled Hybrid Endocrowns With Variable Preparations

**DOI:** 10.1111/jerd.13453

**Published:** 2025-03-03

**Authors:** Izim Turker Kader, Safa Ozer, Burcin Arican

**Affiliations:** ^1^ Bahçeşehir University School of Dental Medicine Department of Prosthodontics Istanbul Turkey; ^2^ Bahçeşehir University Vocational School of Health Services Dental Prosthetic Technology Istanbul Turkey; ^3^ Bahçeşehir University School of Dental Medicine Department of Endodontics Istanbul Turkey

**Keywords:** dental materials, dental prosthesis, dental prosthesis design, three‐dimensional printing

## Abstract

**Objective:**

This study aims to evaluate the influence of different preparation designs on the marginal and internal fit of 3D‐printed permanent endocrowns using circumferential 3D analysis.

**Materials and Methods:**

Maxillary right first molar typodont teeth were prepared with four designs (*n* = 12); Group 1‐butt joint & 2 mm pulp chamber, Group 2‐butt joint & 4 mm pulp chamber, Group 3‐shoulder & 2 mm pulp chamber, and Group 4‐shoulder & 4 mm pulp chamber. Teeth were scanned, replicated as 3D‐printed master dies, and restored with 3D‐printed ceramic‐filled hybrid endocrowns. After seating with Fit Checker, superimposed scans were analyzed circumferentially at standard points. Marginal, internal, pulp chamber, and overall gap values were measured from mesiodistal, buccolingual, and oblique sections. Statistical analysis was performed using two‐way anova and post hoc Tukey tests (*α* = 0.05).

**Results:**

Margin design and pulp chamber depth interactions revealed significant differences (*p* < 0.05). The least marginal fit was in Group 2, while the best internal and overall fit was in Group 3 (*p* < 0.05).

**Conclusion:**

Different preparation designs have an impact on the fit of endocrowns. A shoulder margin design and a 2‐mm pulp chamber depth exhibited a better internal fit of 3D‐printed ceramic‐filled hybrid endocrowns through circumferential 3D analysis.

**Clinical Significance:**

Circumferential 3D analysis reveals that variable preparation designs significantly influence the fit of endocrowns, guiding clinicians in selecting optimal designs for improved clinical outcomes.

## 1 Introduction

Endodontically treated teeth are at a higher risk of fracture due to significant loss of tooth structure. Therefore, the gold standard for treating these teeth should prioritize a minimally invasive approach that preserves as much tooth structure as possible [1]. Consequently, restorative materials should fulfill the mechanical, functional, and aesthetic requirements and obtain a long‐term coronal seal [2].

Endocrowns have become increasingly popular for restoring structurally compromised, endodontically treated teeth [3]. Endocrown, introduced two decades ago, is a monolithic restoration that extends into the pulp chamber, allowing for enhanced retention and using its extensive surface area to establish a robust adhesive bond with the tooth structure [4]. The depth and shape of the pulp chamber, along with the finish line design—whether circumferential butt joint margin and shoulder or chamfer—affect the marginal and internal adaptation of endocrowns [5, 6]. In the literature, endocrown pulp chamber depth preparations range from 2 to 7 mm [7, 8]; however, depths of 2 and 4 mm are more commonly utilized [9, 10].

Poor marginal adaptation can lead to plaque accumulation, increasing the likelihood of secondary caries, periodontal disease, and endodontic complications, potentially affecting the longevity of these restorations [11, 12]. In addition, proper internal adaptation is crucial, particularly for placing all‐ceramic restorations, as it ensures even stress distribution during mastication [13].

Adequate marginal and internal adaptations are important criteria to consider when evaluating the fit of restorations [14]. One method of assessing the adaptation of restorations is using fit‐indicating materials [14, 15]. Fit Checker (GC Europe, Leuven, Belgium), the fit‐indicating material, is a modified polyvinyl siloxane material used specifically to evaluate the marginal and internal fit of the restorations [16]. This elastomeric material provides a clear representation of gaps between the restoration and abutment tooth, enabling precise adjustments. The use of Fit Checker for evaluating the fit of restorations remains insufficiently explored. Existing literature suggests that during the silicone disclosing procedure, Fit Checker interacts with both the restoration's internal surface and the prepared tooth, providing insights into adaptation accuracy [16, 17].

Three‐dimensional (3D) printing technologies are revolutionizing dental restorations by offering superior accuracy, reduced errors, and cost efficiency over traditional methods [18]. These advantages significantly improve the marginal and internal adaptation of restorations, such as endocrowns, leading to better clinical outcomes [19]. Recent advancements have focused on developing diverse 3D‐printed materials, including metals, ceramics, polymers, and composites, to further optimize the precision and adaptability of restorations [18]. Among these, ceramic‐filled hybrid materials have gained attention due to their improved mechanical properties and suitability for various dental applications [20]. VarseoSmile TriniQ (Bego, Bremer, Germany) is a notable example, designed for single crowns, inlays, onlays, anterior and posterior veneers, including occlusal surfaces like endocrowns. According to the manufacturer, this material exhibits favorable mechanical properties, making it a reliable option for permanent restorations (BEGO, VarseoSmile TriniQ Technical Product Information Data Sheet, 2023).

Different methods have been proposed to evaluate the marginal and internal fit of restorations, mainly categorized as two‐dimensional (2D) evaluation or 3D analysis methods [21, 22]. Among these, 3D digital analysis using a fit‐indicating material involves scanning the abutment tooth and then applying fit‐indicating material over it by using intraoral scanners. This approach eliminates the need for sectioning and provides a more comprehensive evaluation of marginal and internal fit by measuring hundreds to thousands of points or measuring the mean gap circumferentially in specific areas of interest, derived from unlimited cross‐sections across all three dimensions [21, 23, 24, 25].

To the best of the authors' knowledge, there are studies in the literature comparing the effects of different preparation designs on the marginal and internal fit of endocrowns only from mesiodistal (MD) and buccolingual (BL) sections by various analysis methods [24, 25, 26, 27]. However, no study has evaluated the adaptation of 3D‐printed ceramic‐filled hybrid endocrowns through circumferential 3D analysis, including MD, BL, oblique 1, and oblique 2 sections. Therefore, the present study aims to evaluate the influence of different preparation designs on the marginal and internal fit of 3D‐printed ceramic‐filled hybrid endocrowns by circumferential 3D analysis. The null hypothesis for this study was that different preparation designs would not significantly affect either the marginal or internal fit of endocrowns when evaluated through circumferential 3D analysis.

## 2 Material and Methods

The sample size calculation was conducted using the G*Power v3.1.9.2 (Heinrich‐Heine‐Universität Düsseldorf, Düsseldorf, Germany) statistical software based on data from a previous study by Gaintantzopoulou et al. [9] A minimum of 12 specimens per group was required to achieve 95% statistical power to detect differences, with a significance level set at 0.05, to test the null hypotheses.

### 2.1 Pilot Study

Prior to the present study, a pilot study was carried out with two samples from each group. During the pilot study, one of the authors (S.Ö.) gained experience by completing the final preparations after several preparation trials using a dental microscope (Zumax OMS 2000, Zumax, China) and scanning the samples with a digital intraoral scanner (CEREC AC, Primescan, Dentsply Sirona, York, PA, USA). Master dies were designed and fabricated both as single units and in sets of four. A preference was given for fabricating all prepared samples as single master dies due to ease of measurement and because the results showed variation in sets of four. Endocrowns adhered to 3D‐printed models using Fit Checker silicone fit‐indicating and light‐body impression material. However, since the Fit Checker material showed better adhesion to the surface, it was selected. Additionally, measurement difficulties were encountered in the master dies in sets of four due to their rapid setting. To ensure easy removal of the endocrowns from the model surface, different substances like water, Vaseline, gel, and hand lotion (Geistlich Pharma AG, Bahnhofstrasse, Wolhusen) were tested, with hand lotion providing the best and most consistent results. None of the samples or data from the pilot study were used in the present study. Based on these findings, the main study proceeded as outlined below.

### 2.2 Model Preparations

**FIGURE 1:**
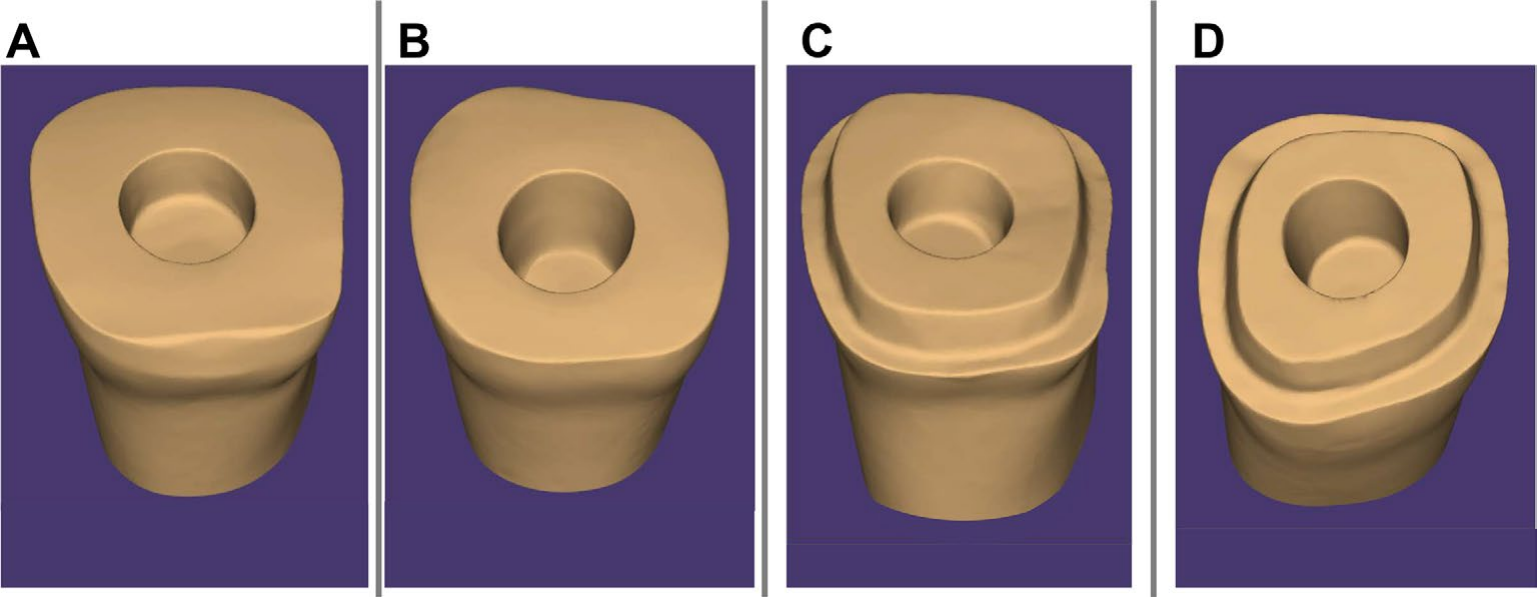
Scans of different preparation designs for Group 1 (A), Group 2 (B), Group 3 (C), and Group 4 (D) selected in exoCAD Dental CAD software.

Four typodont maxillary right first molars (AG‐3 ZE, Frasaco GmbH, Germany) were prepared according to different preparation designs (Figure 1) by one experienced operator (S.Ö.) as follows:

- Group 1: Butt joint preparation and a 2‐mm pulp chamber depth (*n* = 12)
- Group 2: Butt joint preparation and a 4 mm pulp chamber depth (*n* = 12)
- Group 3: 1 mm shoulder preparation and a 2 mm pulp chamber depth (*n* = 12)
- Group 4: 1 mm shoulder preparation and a 4 mm pulp chamber depth (*n* = 12)

All preparations were performed under the dental operation microscope at ×18.4. The preparation for each group commenced with a 2 mm occlusal reduction using a green belt occlusal‐reduction diamond bur (Frank Dental GmbH D.828.017.G.FGA, Gmund, Germany). In Group 1 and Group 2, a green belt wheel diamond bur (Meisinger 909G‐031‐FG Coarse 5/Pk, Neuss, Germany) is used to create a circumferential butt joint margin with a width of 2 mm. Then, a red belt conical diamond bur (Frank Dental GmbH D.845KR.016.G.FGA, Gmund, Germany) with an internal taper of 8° axial walls is used to prepare the pulp chamber [28]. A red belt medium round‐end tapered diamond bur (Frank Dental GmbH D.850.016.FG, Gmund, Germany) was also used to round down the internal line angle, remove irregularities, and produce a flat polished surface. In Group 3 and Group 4, the entire preparation procedure was performed with the same burs used in Group 1 and Group 2. Unlike the first two groups, after occlusal reduction with an occlusal‐reduction diamond bur and pulp chamber preparation with a conical diamond bur, a red belt modified shoulder fine W diamond bur (Meisinger 848WF‐018‐FG, Gmund, Germany) was used in 1 mm shoulder margin preparation. After the preparations, occlusal reductions, margin widths, and pulp chamber depths were measured by a periodontal probe and verified by a digital caliper (Digimatic Micrometer Mitutoyo Corp., Japan).

### 2.3 The Fabrication of Master Dies

For each group, one prepared typodont molar tooth was scanned using the digital intraoral scanner, and the external CAD data was processed using a dental laboratory scanner (InEos X5, Dentsply Sirona, York, PA). Standard tessellation language (STL) files were acquired and processed in Shapr 3D (Budapest, Hungary), a CAD software used to design and create ready‐to‐manufacture models, with a base drawn under the preparation scans. The prepared molars were then replicated into single master dies using a digital light processing 3D printer (Asiga Ultra (50), ASIGA, Sydney, Australia) and 3D‐printed model resin (VarseoWax Model, Bego, Bremer, Germany) in the horizontal direction (0^0^) with a layer thickness of 50 μm. After printing, the dies were washed in 99% isopropanol alcohol for 3 min (Form Wash, Formlabs, Somerville, USA) and post‐cured twice for 20 min at 60°C (Form Cure, Formlabs, Somerville, USA) following the manufacturer's instructions.

### 2.4 Endocrown Design

For the endocrown design, typodont maxillary molar teeth were scanned before and after preparation. The resulting STL data were processed using exocad DentalCAD software (exocad GmbH, Darmstadt, Germany). Endocrown designs were created based on the prepared tooth STLs to reflect the initial form of the tooth. During the chairside CAD design process, the cement space was set at 80 μm. The endocrowns were then manufactured horizontally (0°) using 3D‐printed ceramic‐filled hybrid material (VarseoSmile Triniq, Bego, Bremer, Germany) with a layer thickness of 50 μm. After printing, the endocrowns were washed in 99% isopropanol alcohol for 5 min (Form Wash, Formlabs, Somerville, USA) and post‐cured twice for 20 min at 60°C (Form Cure, Formlabs, Somerville, USA), following the manufacturer's recommendations.

### 2.5 Endocrowns Placement

A 3D analysis technique using a 3D software program (OraCheck, Cyfex AG, Zurich, Switzerland) was performed to evaluate fit. The endocrown preparation dies were first scanned using the digital intraoral scanner and saved as the master digital file of the preparation. The inner surface of each endocrown was lightly wiped with a lubricant (hand lotion) before injecting a thin layer of Fit Checker silicone fit‐indicating material (GC Europe, Leuven, Belgium). Each endocrown was loaded and seated for 2 min to obtain the complete setting time under a constant axial force of 50 N [29]. 5 kg weight was used to standardize the applied force, corresponding to 50 N. Excess silicone material was removed from the margins under the dental microscope using a no. 12 surgical blade (Feather Safety Razor Co. Ltd., Osaka, Japan).

### 2.6 Determination of Measurement Points and Circumferential Gap Analysis

**FIGURE 2:**
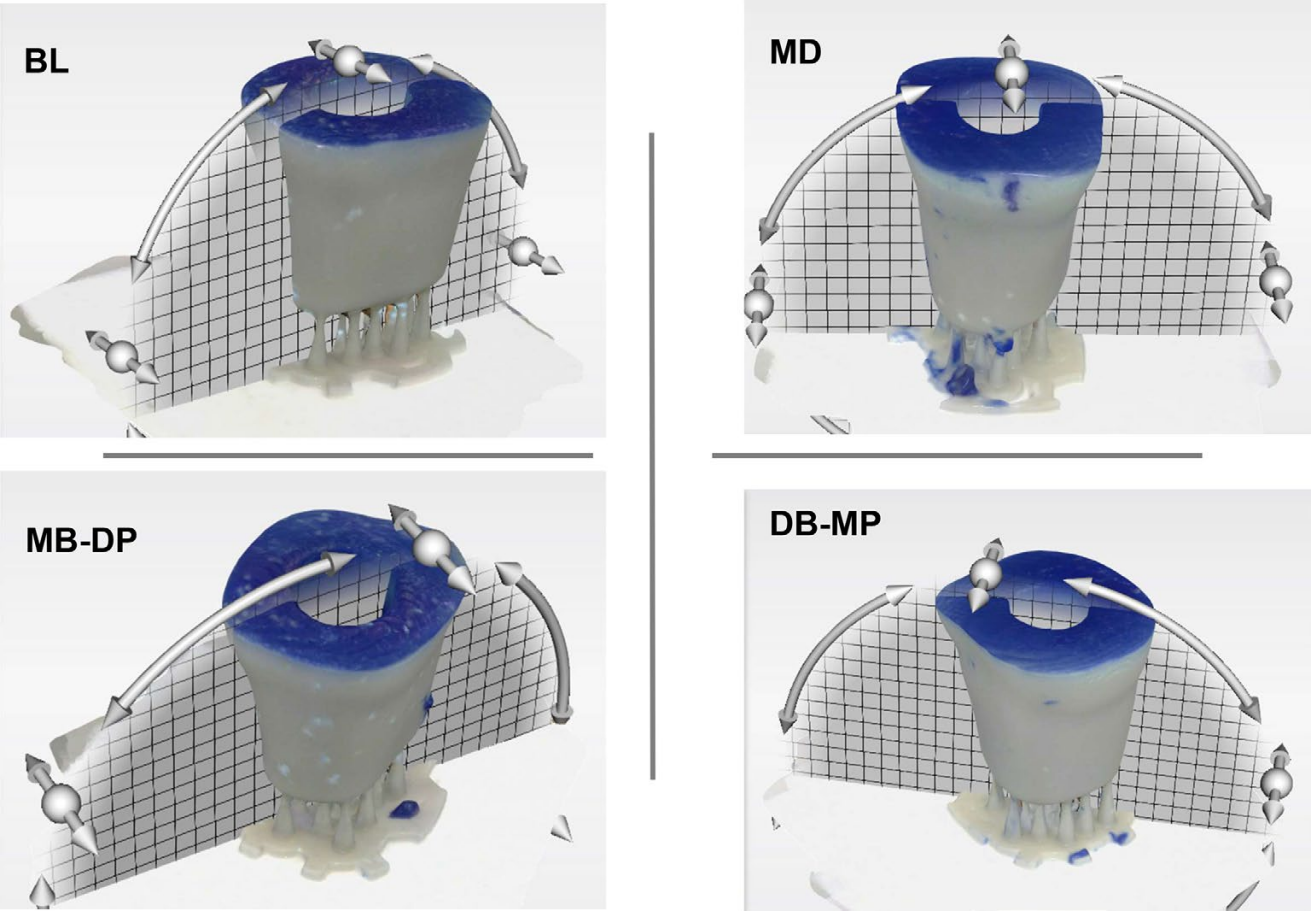
The buccolingual (BL), mesiodistal (MD), oblique 1 (mesiobuccal (MB)—distopalatinal (DP)), and oblique 2 (distobuccal (DB)—mesiopalatinal (MP)) sections chosen in the Oracheck 3D analysis software from one of the superimposition views.

After removing the endocrown from the preparation, a second scan with the impression material covering the preparation die was performed using the digital intraoral scanner. The 3D analysis software program digitally superimposed the two recorded scans in STL files for each tested group. The subtractive analysis was accomplished by calculating the distances from each surface point of the first data set to the surface points of the second data set. The software's best‐fit algorithm selected approximately 20,000 points per surface matching [24]. The vertical sections were selected from the core region of each superimposition in the buccolingual (BL), mesiodistal (MD), and oblique 1 (mesiobuccal (MB)‐distopalatinal (DP)), and oblique 2 (distobuccal (DB)‐mesiopalatinal (MP)) sections to evaluate the circumferential fit (Figure 2).

**FIGURE 3:**
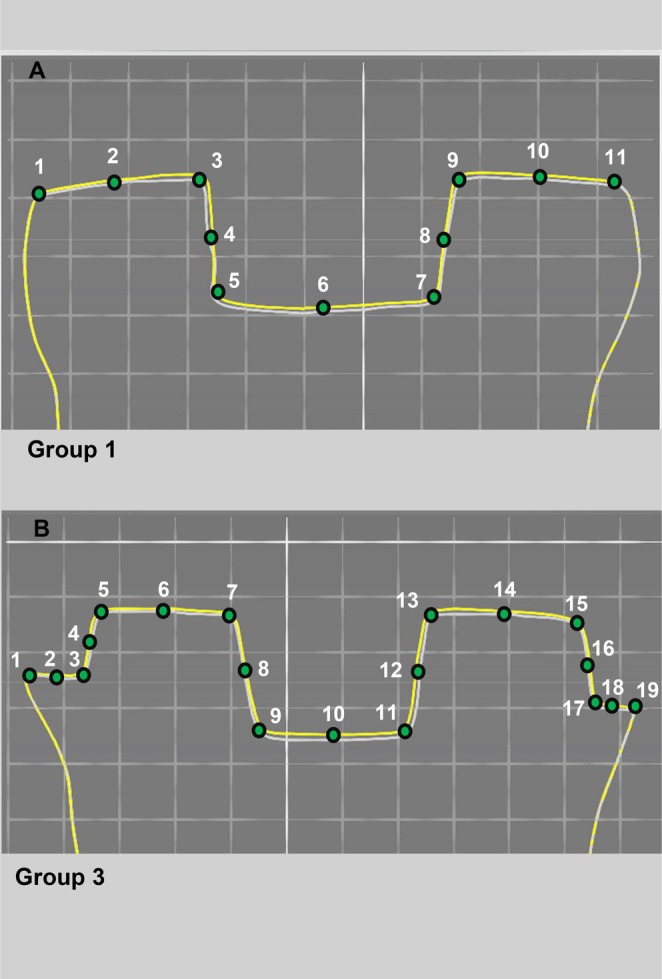
(A) Marginal and internal fit analysis of Group 1 from 11 points in the image taken via STL data superimposition in Oracheck 3D analysis software. The mean of points 1 and 11 for marginal gap (MG), 2–10 for internal gap (IG), 3–9 for pulp chamber gap (PCG), and 1–11 for overall gap (OG) measurements. (B) Marginal and internal fit analysis of Group 3 from 19 points in the image taken via STL data superimposition in Oracheck 3D analysis software. The mean of points 1 and 19 for MG, 2–18 for internal gap IG, 7–13 for PCG, and 1–19 for OG measurements. Images represent mesiodistal (MD) sections. Each square is 1 mm^2^.

**FIGURE 4:**
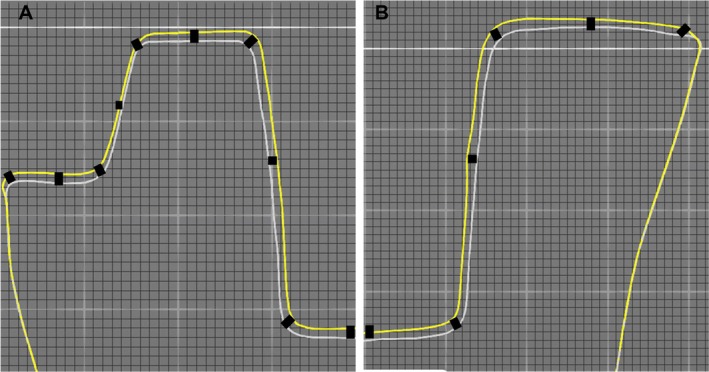
(A) The measurement performed at relevant points in the close‐up image of Group 4. (B) the measurement performed at relevant points in the close‐up image of Group 2.

Gap measurement points are shown in Figure 3 on the superimposition views of Group 1 and Group 3. Notably, the points assessed for the pulp chamber gap are included in the internal gap measurements. The overall gap assessment encompasses both marginal and internal gap regions. Detailed cross‐sectional close‐ups of the measurement points are shown in Figure 4.

### 2.7 Statistical Analysis

The data were analyzed using IBM SPSS V23 (IBM Statistics, Armonk, NY) and Minitab V14 (Minitab LLL, State College, Pennsylvania). The normality of distribution was examined with the Shapiro–Wilk Test. A Two‐Way ANOVA was employed to compare measurements that did not follow a normal distribution according to the preparation designs, and multiple comparisons were made with the Tukey test. The relationship between the quantitative data that followed a normal distribution was examined with the Pearson rho correlation coefficient. The analysis results were presented as mean ± standard deviation. The significance level was set as *p* < 0.05.

## 3 Results

Based on the data, marginal gap (MG), internal gap (IG), pulp chamber gap (PCG) and overall gap (OG) measurements were compared according to different preparation designs and pulp chamber depths. No significant differences were found between groups in terms of PCG values (*p* > 0.05), whereas statistically significant differences were observed in the MG, IG, and OG values (*p* < 0.05). The pulp chamber depth, preparation design, and interaction between the two were compared statistically, and the results are shown in Table 1. The results of multiple comparisons according to gap measurements of different preparation designs and pulp chamber depth data by groups are presented in Table 2.

**TABLE 1:** Comparison of marginal gap (MG), internal gap (IG), pulp chamber gap (PCG) and overall gap (OG) values according to pulp chamber depth and preparation design.

	*F*	*p*	$\;\eta^{2}$
MG measurements			
Pulp chamber (PC) depth	1.227	0.274	0.028
Preparation design	60.984	0.000	1.389
PC depth * preparation design	46.001	0.000	1.047
IG measurements			
Pulp chamber (PC) depth	1.508	0.226	0.033
Preparation design	35.223	0.000	0.445
PC depth * preparation design	14.41	0.0004	0.247
PCG measurements			
Pulp chamber (PC) depth	0.664	0.420	0.015
Preparation design	7.23	0.008	0.149
PC depth * preparation design	3.563	0.066	0.075
OG measurements			
Pulp chamber (PC) depth	0.591	0.446	0.013
Preparation design	14.876	0.0004	0.253
PC depth * preparation design	21.954	0.000	0.333

*Note*: The significance level was set as *p* < 0.05.

Abbreviations: *F* = two‐way anova test statistic; $\;\eta^{2}$ = partial eta square.

**TABLE 2:** Two‐way anova results for the mean marginal gap (MG), internal gap (IG), pulp chamber gap (PCG) and overall gap (OG) values (mm) of different groups.

MG measurements			
Preparation design	Pulp chamber depth		***p***
	2 mm PC	4 mm PC	
Butt joint	0.034 ± 0.008^b^	0.019 ± 0.005^c^	< 0.001
Shoulder	0.036 ± 0.007^b^	0.047 ± 0.006^a^	
IG measurements			
Preparation design	Pulp chamber depth		
	2 mm PC	4 mm PC	
Butt joint	0.084 ± 0.009^a^	0.078 ± 0.008^ab^	< 0.001
Shoulder	0.062 ± 0.006^c^	0.073 ± 0.006^b^	
PCG measurements			
Preparation design	Pulp chamber depth		
	2 mm PC	4 mm PC	
Butt joint	0.082 ± 0.009	0.079 ± 0.008	0.066
Shoulder	0.072 ± 0.007	0.078 ± 0.005	
OG measurements			
Preparation design	Pulp chamber depth		
	2 mm PC	4 mm PC	
Butt joint	0.075 ± 0.009^a^	0.067 ± 0.007^b^	< 0.001
Shoulder	0.058 ± 0.005^c^	0.069 ± 0.005^ab^	

*Note*: Mean ± Standard deviation; Different superscript letters (a, b, c) indicate statistically significant differences within the same column, while identical letters denote no significant difference. Statistically significant at *p* < 0.05.

Abbreviation: PC, pulp chamber.

**FIGURE 5:**
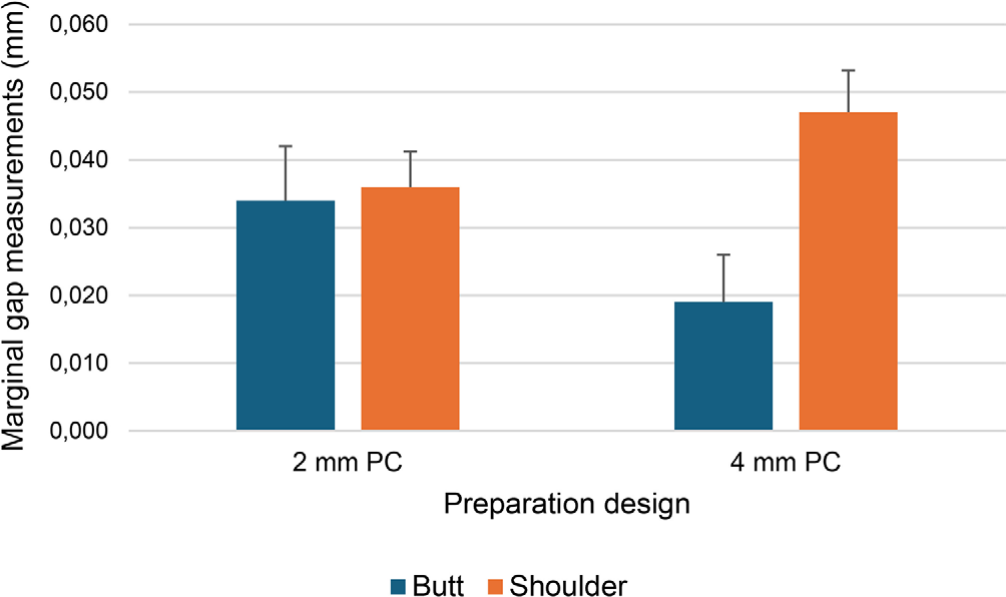
The column chart of marginal gap (MG) measurements of groups with different preparation designs.

When comparing preparation designs in terms of MG values, the butt joint preparation design had a significantly lower MG value compared to the shoulder preparation design (*p* < 0.05) (Table 1). While the lowest MG value was obtained with the butt joint preparation design and a pulp chamber depth of 4 mm (Group 2), the highest MG value was identified in the shoulder preparation design and a pulp chamber depth of 4 mm (Group 4) (*p* < 0.05) (Table 2) (Figure 5).

**FIGURE 6:**
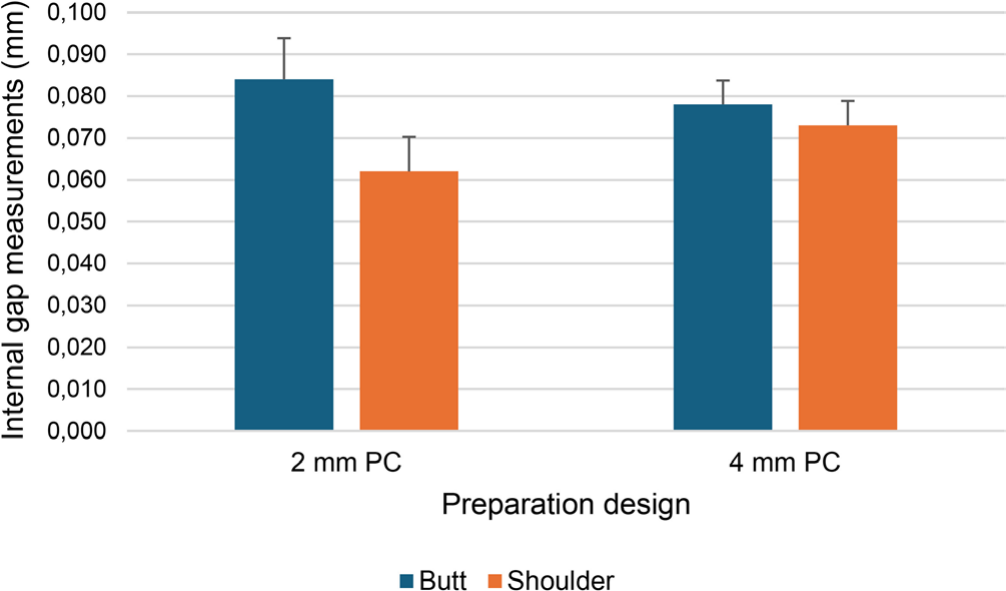
The column chart of internal gap (IG) measurements of groups with different preparation designs.

Statistically significant differences were detected both within preparation design groups and interactions between groups when comparing IG values (*p* < 0.05) (Table 1). The highest gap value was identified in the butt joint preparation design and a pulp chamber depth of 2 mm (Group 1), while the lowest IG value was obtained in the shoulder design and a pulp chamber depth of 2 mm (Group 3) (*p* < 0.05) (Table 2) (Figure 6).

Regarding the preparation design regarding PCG values, the shoulder preparation design showed a lower value than the butt joint design (*p* < 0.05) (Table 2). Additionally, statistically significant values were obtained when comparing OG values (*p* < 0.05) (Table 1). The highest OG value belonged to the butt joint preparation design and a pulp chamber depth of 2 mm (Group 1), and the lowest gap was identified in the shoulder preparation design and a pulp chamber depth of 2 mm (Group 3) (*p* < 0.05) (Table 2).

## 4 Discussion

Maintaining an acceptable fit between endocrowns and their abutments, both in terms of marginal and internal gaps, is essential for achieving favorable long‐term results [30]. This study evaluated the influence of different preparation designs on the marginal and internal fit of 3D‐printed permanent endocrowns by circumferential 3D analysis. Based on the comparative evaluation of marginal and internal fit, the null hypothesis was rejected because different preparation designs showed significant differences in the marginal and internal fit of 3D‐printed ceramic‐filled hybrid endocrowns.

Adaptation can be evaluated using various methods, including 3D analysis [24, 25]. While 2D methods are the most employed techniques for assessing the marginal and internal gaps of restorations [26, 27], this study utilizes 3D analysis software to achieve more precise evaluations without data loss, covering unlimited measurement points across all three dimensions. The selection of the measurement points is crucial in adaptation assessment. Groten et al. [12] reported that a minimum of 10 points should be randomly selected to measure the fit of the restoration, and measurements should be performed throughout the restoration [31]. Additionally, Gassino et al. [32] stated that measurements should be performed all around the crown. In the present study, measurements were performed from 11 points in butt joint and 19 points in the shoulder preparation design for the fit assessment by a circumferential 3D analysis of mesiodistal (MD), buccolingual (BL), oblique 1, and oblique 2 cross‐sections joining the endocrown corners for measurement, which enabled the higher reliability of our results.

Different preparation designs can influence the marginal and internal adaptation of endocrowns [33]. Farghal et al. [34] reported that butt joint preparation design results in better adaptation with less marginal and internal gaps than 1‐ and 2‐mm ferrule (shoulder) preparation, regardless of the ceramic material used. Additionally, the shoulder preparation design was found to be more retentive. Seo et al. [35] also noted that endocrowns with a 1 mm shoulder finish line offer a larger surface area for bonding, as this design incorporates four axial walls instead of two, as seen in butt joint preparations. Moreover, they highlighted that the increased surface area provided by shoulder margins enhances marginal adaptation. However, in accordance with the study of Farghal et al. [34] the butt joint design exhibited the best marginal fit, whereas the shoulder preparation design showed the poorest in this study. This might be related to the fit‐indicating material finding a more convenient escape way since there are fewer axial walls in the butt joint design. Additionally, variations in pulp chamber depth were considered a potential factor, though no significant differences were found between the 2 and 4‐mm depths in this study.

Different endocrown preparation depths assume that a deeper pulpal cavity provides more surface for bonding, better load transmission, and stabilization during cementation [36]. Some studies confirmed that the increase in cavity depth of endocrowns leads to a significant increase in the marginal gap [9, 10]. Gaintantzopoulou et al. [9] concluded that the increased intraradicular extension of endocrowns resulted in higher marginal and internal gaps with a negative effect on their adaptations. Although this study found no significant differences between 2 and 4 mm pulp chamber depths, notable differences were observed when comparing the groups. Group 2 exhibited the least marginal gap, while Group 4 had the largest, possibly due to the fit‐indicating material flowing through the butt joint margins, which have only two axial walls. The lowest internal and overall gaps were observed in Group 3, while the highest were in Group 1, likely due to the possible slight movement of the restoration during seating in Group 1.

The mechanical properties of the restoration in terms of retention are significantly influenced by the internal fit [36]. To analyze internal fit in greater detail, the measurements of the pulp chamber gap were also performed in this study for better comparisons. According to Taha et al. [11] shoulder finish line enhances better force distribution, decreasing the load transmitted to the pulpal floor and reducing resin cement thickness. This might provide better distribution of the cement. Inconsistent with the mentioned study, although there was no significant difference in different pulp chamber depths in this study, it was observed that there was less pulp chamber gap in the shoulder preparation design compared to the butt joint. Unlike the study by Farghal et al. [34] mentioned above, in this study, a better internal fit was obtained in the shoulder preparation design compared to the butt joint. This might be because the shoulder preparation design, which was found to be more retentive in their study, created mechanical locking when placed, allowing the endocrown restoration a better seating and improved internal fit. Additionally, while the mentioned studies focused on milled materials, using a 3D‐printed ceramic‐filled hybrid material in this study may have improved internal fit.

3D‐printed materials are increasingly studied for their accuracy, adaptation, and clinical performance, often compared to milled alternatives [18]. While some studies report superior marginal adaptation and internal fit for 3D‐printed ceramic‐filled hybrid and interim crowns [37, 38, 39], others, such as Dewan et al. [40] found that milled zirconia crowns exhibited higher marginal integrity. A systematic review concluded that the marginal and internal fit were generally comparable between 3D‐printed and milled ceramics and polymers, with material composition playing a crucial role [18]. It has been reported that 3D‐printed ceramics may exhibit lower accuracy than polymer‐based structures due to their crystalline content [41]. However, the lower ceramic content in hybrid materials compared to all‐ceramics or polycrystalline zirconia may enhance their fit, particularly in endocrown restorations. A prospective clinical study demonstrated acceptable marginal adaptation of 3D‐printed ceramic‐filled hybrid endocrowns after 2 years [42]. Despite these promising findings, further in vitro studies are needed to assess mechanical properties and long‐term clinical performance.

A pilot study was conducted beforehand, and the methodology is based on the results obtained to strengthen the validity of this study. To standardize measurements, provide high clinical relevance, and eliminate variations in natural teeth size and shape, typodont teeth were used, with all preparations performed by a single operator after multiple trials. Fit Checker, an alternative fit‐indicating material specifically designed to evaluate the marginal and internal fit of restorations, was used in this study because light‐body impression material often remained inside the endocrown rather than on the master die and tended to tear more easily. A circumferential 3D analysis method, which involves the measurement of the gap by overlapping a scanned tooth surface and impression surface, is used to perform a more accurate analysis without data loss on measurement points. Simultaneously, with the aid of circumferential analysis, oblique measurements were performed, expanding the evaluation beyond just two sections.

This in vitro study has several limitations. Only one type of digital intraoral scanner, 3D printer, and 3D‐printed material was utilized, which may limit the generalizability of the findings. Future studies incorporating various scanners, printers, and materials could provide more comprehensive insights and strengthen the evidence base. Additionally, the restorations in this study were not cemented to allow for precise gap measurements; however, this may have contributed to the increased overall gap values. Including the cementation process and related evaluations in future studies would enhance the clinical relevance of the findings. Furthermore, this study employed only Fit Checker as a fit‐indicating material. Comparative evaluations using alternative materials, such as light‐body silicone, are recommended to further validate the results. Lastly, while this study provides valuable in vitro data, in vivo studies are essential to confirm these findings under clinical conditions and to assess the performance of endocrowns in real‐world scenarios. Addressing these limitations in future research will further advance the understanding of endocrown restorations.

## 5 Conclusion

Within the limitations of this study, it was concluded that:

A butt joint preparation with a 4 mm pulp chamber depth provided a superior marginal fit for 3D‐printed endocrown restorations. However, a butt joint preparation and a 2 mm pulp chamber depth resulted in inferior internal fit compared to the shoulder preparation and a 2 mm pulp chamber depth. For clinical applications of 3D‐printed ceramic‐filled hybrid endocrowns, a 1 mm shoulder preparation and a 2 mm pulp chamber depth are recommended for optimal fit.

## Conflicts of Interest

The authors declare no conflicts of interest.

## Data Availability

The data that support the findings of this study are available on request from the corresponding author. The data are not publicly available due to privacy or ethical restrictions.
